# Goji-Berry-Mediated Green Synthesis of Gold Nanoparticles and Their Promising Effect on Reducing Oxidative Stress and Inflammation in Experimental Hyperglycemia

**DOI:** 10.3390/antiox12081489

**Published:** 2023-07-25

**Authors:** Luminita David, Valentina Morosan, Bianca Moldovan, Gabriela Adriana Filip, Ioana Baldea

**Affiliations:** 1 Faculty of Chemistry and Chemical Engineering, “Babeş-Bolyai” University, 11 Arany Janos Street, 400028 Cluj-Napoca, Romania; luminita.david@ubbcluj.ro (L.D.); valentina.morosan@ubbcluj.ro (V.M.); 2 Department of Physiology, “Iuliu Haţieganu” University of Medicine and Pharmacy, 1-3 Clinicilor Street, 400006 Cluj-Napoca, Romania; adrianafilip33@yahoo.com (G.A.F.); baldeaioana@gmail.com (I.B.)

**Keywords:** green synthesis, goji berries, gold nanoparticles, antioxidant activity

## Abstract

The present report focuses on a rapid and convenient method applicable in the green synthesis of gold nanoparticles (AuNPs) using goji berry (*Lycium barbarum*—LB) extracts rich in antioxidant compounds, as well as on the structural analysis and evaluation of the induced antioxidant protection and anti-inflammatory effects of the synthesized gold nanoparticles upon endothelial cells (HUVECs) exposed to hyperglycemia. The synthesized AuNPs were characterized using ultraviolet–visible (UV-Vis) spectroscopy and transmission electron microscopy (TEM), whereas the presence of bioactive compounds from the *L. barbarum* fruit extract on the surface of the nanoparticles was confirmed using Fourier transform infrared spectroscopy (FTIR). The antioxidant activity of the biosynthesized gold nanoparticles was evaluated on the HUVEC cell line. The results reveal that AuNPs with a predominantly spherical shape and an average size of 30 nm were obtained. The UV-Vis spectrum showed a characteristic absorption band at λ_max_ = 536 nm of AuNPs. FTIR analysis revealed the presence of phenolic acids, flavonoids and carotenoids acting as capping and stabilizing agents of AuNPs. Both the *L. barbarum* extract and AuNPs were well tolerated by HUVECs, increased the antioxidant defense and decreased the production of inflammatory cytokines induced via hyperglycemia-mediated oxidative damage.

## 1. Introduction

Nanotechnology is nowadays one of the most dynamic research fields of materials science. Among nanomaterials, metallic nanoparticles are widely used due to their specific properties, which confer them a large range of applications in various fields, such as optics, electronics, environmental remediation, catalysis, cosmetics and medicine [1,2]. Their large variety of utilizations has strongly increased interest in developing synthesis strategies to obtain high-purity, highly stable, monodisperse, innocuous and affordable metallic nanoparticles. The conventional chemical (chemical reduction and the sol–gel method) and physical (thermal decomposition and laser ablation) synthetic approaches generally involve the use of hazardous chemicals; require high energy and complex equipment; are labor-intensive; and deliver polydisperse, non-uniform, unstable and harmful nanoparticles. Therefore, searching for alternative green synthetic methods has become a primary concern. This has led to the substantial development of biological approaches for obtaining metallic nanoparticles methods that prevent the use of harmful chemicals and are reliable, cost-effective, easy to scale up and environmentally friendly. Furthermore, the biosynthesized nanoparticles usually possess enhanced stability and biocompatibility with no harmful effects. This makes them suitable for pharmaceutical and food applications [2,3,4,5]. The green strategies for the synthesis of metal nanoparticles exploit the tremendous potential of the biomass as a source of reducing and stabilizing agents for the metallic nanomaterials. To this end, various microorganisms and plants have been successfully used as nano-biofactories. The microbial-assisted synthesis of nanoparticles usually requires laborious purification in order to remove potential pathogenic or toxic compounds, especially when the nanoparticles are intended for biological applications. As a consequence, plant extracts rich in bioactive compounds such as amino acids, saccharides, terpenoids, polyphenols, flavonoids and alkaloids are by far the most investigated and applied agents in the green synthesis of metallic nanoparticles. Many plants have been noted for their ability to synthesize both silver and gold nanoparticles with biomedical applications, such as tea plant [6], milk thistle [7], cornelian cherry [8], European cranberry bush [9] and sage [1].

Goji berries (*L. barbarum* L.) are fruits well known for their high antioxidant potential. Goji berries are used in traditional Chinese medicine because of their numerous health benefits, including their ability to treat hypertension, infectious diseases, common illnesses and diabetes, as well as to regulate cholesterol and triglyceride levels; prevent tumors, aging effects and heart disease; and reduce anxiety, depression, fatigue and stress. All of these beneficial effects are mostly conferred by the presence of strong antioxidant compounds in these fruits, such as polyphenols, flavonoids, carotenoids and vitamins [10,11].

Because it is well documented that all these compounds possess a remarkable ability to reduce metal ions and to cap and stabilize the obtained zero valent metal nanoparticles, we decided to exploit these strong antioxidant fruits for the biosynthesis of biocompatible gold nanoparticles. To the best of our knowledge, this is the first report on the use of *L. barbarum* fruits’ bioactive compounds for the green synthesis of AuNPs. Hence, the current study aimed to synthesize, characterize and investigate the antioxidant effect of gold nanoparticles in a biological environment against hyperglycemia-induced oxidative stress.

In diabetic patients, continuous or intermittent hyperglycemia generates free radical species through the protein kinase C (PKC) and NADPH oxidase pathways and involves mitochondrial respiratory chain enzymes, causing cytokine release and further inflammation, lack of angiogenesis control and cell death, which leads to complications such as retinopathy, neuropathy, nephropathy and cardiovascular diseases [12,13]. Moreover, hyperglycemia can induce the formation of advanced glycation end products (AGEs) that activate NF-kB and the transcription of proinflammatory genes [14]. The synthesis of cytokines, TGF-β, chemokines and vascular cell adhesion molecules (VCAMs) is responsible for the induction of endothelial cell inflammation and apoptosis [15]

There is a lot of research in this field with the aim of finding antioxidant agents that can interrupt these metabolic alterations in diabetes in order to delay the onset and evolution of ROS-induced complications.

The aim of this biological study was to find the possible beneficial effects of gold nanoparticles phytosynthesized using *L. barbarum* extract against high-glucose-environment-induced oxidative stress and inflammatory status. Therefore, here, we report the protective antioxidant and anti-inflammatory effects against hyperglycemia-induced oxidative stress by AuNPs and *L. barbarum* extract in vitro on endothelial cells with respect to oxidative-stress-induced lipid peroxidation antioxidant defense mechanisms and the synthesis of proinflammatory cytokines interleukin 1 α (IL1α) and interleukin 1β (IL1β).

## 2. Materials and Methods

### 2.1. Chemical Assays

#### 2.1.1. Reagents

Tetrachloroauric acid trihydrate, sodium hydroxide, Folin–Ciocalteu reagent, 2,2’-azino-bis(3-ethylbenzothiazoline-6-sulfonic acid) (ABTS), 2-thiobarbituric acid and Trolox gallic acid were obtained from Merck (Darmstadt, Germany). Cytochrome c and *o*-phtalaldehyde were obtained from Sigma Aldrich, St. Louis, MO, USA.

#### 2.1.2. Preparation of Goji Fruit Extract

*L. barbarum* L. (red goji) berries were obtained from a small ecological farm, “Goji Bio Oltenia”, in Oltenia province (Romania). Fully ripened fruits of *L. barbarum*, Transilvania cultivar, were harvested in September 2022 in SV Romania, Bradesti (44°30′17″ N, 23°37′13″ E), Dolj County. The plant sample collection required no specific permissions, as it did not involve protected or endangered species. A voucher specimen (FCIC.2022.G1) was deposited in the Department of Chemistry, Babes-Bolyai University, Cluj-Napoca, Romania.

Two grams of powder-ground dried berries was subjected to successive extractions using 40 mL double-distilled water twice. After 2 h at 20 °C, the mixture was filtered through nr. 1 Whitman paper, and the obtained extract was stored at 2 °C until further analysis and use.

The phenolic content of the goji berry extract was evaluated using a slightly modified Folin–Ciocalteu method [16]. Thus, 1.2 mL of a 0.7 M sodium carbonate solution was added to a mixture of 250 μL of goji extract and 1.5 mL of Folin–Ciocalteu reagent, which was incubated for 5 min in the dark. The sample was allowed to react for 2 h at room temperature in the absence of light, and further, its absorbance was spectrophotometrically monitored at 765 nm. The total phenolic content of the extract was expressed in mg gallic acid equivalents/L using a calibration curve of the reference.

#### 2.1.3. Preparation of Gold Nanoparticles Using the Green Synthetic Method

The goji berry extract obtained as described above was further employed in the green synthesis of gold nanoparticles. In a conical flask, 10 mL of 10-fold diluted fruit extract was treated with a 1 N NaOH solution until pH = 5.5 and then added to 10 mL of boiling HAuCl_4_ 1 mM aqueous solution freshly prepared from tetrachloroauric acid trihydrate and distilled water. The reduction of Au^3+^ ions was visually confirmed by a color change from faint orange to dark purple-red. The goji berry phytocompounds were removed from the gold colloidal solution via centrifugation at 10,000 rpm for 60 min, followed by AuNPs’ purification via pellet washing with deionized water and their resuspension via 10 min of sonication. The purified AuNPs were characterized using appropriate techniques and further used for biological assays.

#### 2.1.4. Characterization of Green Synthesized AuNPs

UV–visible (UV-vis) spectroscopy was the first technique applied to monitor the synthesis of the gold nanoparticles. The UV-vis spectrum was recorded using a Perkin Elmer Lambda 25 spectrometer by scanning the absorbance in the range of 300–800 nm in a quartz cuvette of 1 cm using distilled water as a blank. The green synthesized AuNPs were morphologically characterized via transmission electron microscopy (TEM—using a Hitachi H-7650 automatic transmission electron microscope) at 120 kV on a carbon-coated copper grid. The mean diameter of the AuNPs was determined from the TEM images, using ImageJ 1.53t software, from at least 50 nanoparticles. The presence of the bioactive compounds from the goji extract on the surface of the AuNPs was confirmed by Fourier transform infrared spectroscopy (FTIR) using a Bruker Vector 22 FTIR spectrometer (Brucker, Rosenheim, Germany), in a KBr pellet, in the range of 4000–500 cm^−1^. The hydrodynamic diameter and zeta potential of the biosynthesized AuNPs were assessed using a Zetasizer Nanoseries compact scattering spectrometer (Malvern Instruments Ltd.; Malvern, UK).

#### 2.1.5. In Vitro Antioxidant Activity Assessment

The radical scavenging capacity of the goji extract and the green synthesized AuNPs was assessed using the ABTS^·+^ assay [17]. A volume of 3 mL of freshly prepared and properly diluted (absorbance at 734 nm 0.4–0.7) ABTS^·+^ solution was mixed with 50 μL of extract and stored for 15 min in the dark. The absorbance of the resulting sample was recorded spectrophotometrically at 734 nm, and the obtained values were converted to mM Trolox equivalents in order to express the antioxidant capacity of the samples.

The antioxidant ability of the fruit extract was also assessed using the FRAP assay. The FRAP reagent was prepared according to the method described by Benzie and Strain [18] using 1.25 mL of 0.3 M acetate buffer solution with pH = 3.6, 1.25 mL of 0.01 M TPTZ (2,4,6-tris (2-pyridyl)-s-triazine) solution and 1.25 mL of 0.02 M aqueous solution of iron chloride FeCl_3_·6H_2_O. To 2850 μL of freshly prepared FRAP reagent heated to 37 °C, 150 μL of diluted extract (1/10, *v*/*v*) of goji berries was added, and the mixture was kept in the dark for 30 min. The absorbance of the resulting sample was measured at 593 nm, and the results were expressed in μM Trolox using a calibration curve of the standard.

### 2.2. Biological Assays

#### 2.2.1. Cell Source

Human umbilical vein endothelial cells (HUVEC-ECCAC obtained from Sigma Aldrich, Steinheim am Albuch, Germany) and dermal fibroblasts (BJ CRL-2522^™^—purchased from ATCC, VA, USA) were cultured in RPMI and, respectively, Dulbecco’s modified Eagle medium (DMEM) supplemented with 5% fetal calf serum, 50 µg/mL gentamicin and 5 ng/mL amphotericin, all procured from Biochrom AG (Berlin, Germany). Cultures were fed twice weekly and incubated in standard cell culture conditions. For experiments, the concentration of 2% FCS in medium was used.

#### 2.2.2. Viability Assay

Cell viability was assessed using the CellTiter 96^®^ AQueous Non-Radioactive Cell Proliferation Assay (Promega Corporation, Madison, WI, USA). Fibroblasts and endothelial cells at a density of 10^4^/wells in 96-well plaques (TPP, Transadingen, Switzerland) were allowed to settle for 24 h and then exposed for 24 h to *L. barbarum* extract and, respectively, AuNPs in different concentrations, 0–70 µg/mL of polyphenols in *L. barbarum* extract or 0–70 µg/mL of AuNPs, suspended in medium.

High glucose exposure was induced by the supplementation of each cell culture medium with glucose up to 4.5 g/L. Cells in hyperglycemia conditions were treated with *L. barbarum* extract and AuNPs in similar concentrations [19].

Viability was measured colorimetrically using an ELISA plate reader (Tecan, Männedorf, Switzerland) set at 540 nm. All experiments were performed in triplicate. The control sample was represented by cells exposed solely to cell medium. The viability results were calculated as % of the control samples. The concentrations of AuNPs or *L. barbarum* extract which decreased the cell viability to less than 70% of the control were considered toxic. The concentration selected to test the antioxidant and anti-inflammatory effects was 6.5 µg/mL of AuNPs and *L*. *barbarum*, respectively.

#### 2.2.3. Experimental Design

Assessments of inflammatory mediators and oxidative stress generation were performed on endothelial cells in order to assess the possible protective effect of the *L. barbarum* and AuNPs in a prooxidant and proinflammatory hyperglycemia environment. Cells were cultivated at a density of 10^5^/cm^2^ (TPP, Transadingen, Switzerland) for 24 h in medium containing 2% FCS, then exposed for 24 h to *L. barbarum* and AuNPs respectively, with and without (w/wt) hyperglycemia.

Six groups were made: (1) controls exposed to medium (C), (2) cells exposed to *L. barbarum*, (3) cells treated with AuNPs (NP), (4) high glucose exposure (4.5 g/L) (HG), concomitant exposure to (5) high glucose and *L. barbarum* extract (HG + LB) and (6) high glucose and AuNPs (HG + NP). After exposure, supernatant and cells were collected. All the experiments were carried out in triplicate.

#### 2.2.4. Cells Lysis

Cell lysates were obtained as previously reported [20]. The total protein concentration was measured using a DC protein assay kit (Bio-Rad, Hercules, CA, USA) and serum ɣ globulin as standard.

#### 2.2.5. Oxidative Stress Assessment

Malondialdehyde (MDA) was measured spectrophotometrically from cell lysates. Data were expressed as nM/mg protein [20,21].

The enzymatic activity assays of catalase (CAT) and superoxide dismutase (SOD) were determined spectrophotometrically from the cell lysates as previously described [21]. Data are reported as units/mg protein.

Reduced (GSH) and oxidized glutathione (GSSG) were measured fluorometrically from cell lysates, using *o*-phtalaldehyde, as reported. GSH and GSSH concentrations were determined using a standard curve. The results were expressed as nmoles/mg protein [22,23]. All reagents for the oxidative stress assessment were bought from Sigma.

#### 2.2.6. Inflammation

The cytokine levels of IL1α and IL1β were determined from the cell supernatant via ELISA assays using the ElabScience ELISA kits (Houston, TX, USA), according to the producer’s instructions. The results are expressed as pg/mg protein for IL1α and OD/mg protein for IL1β.

### 2.3. Statistical Analysis

Statistical differences between *L. barbarum*, AuNPs and control groups were assessed via one-way ANOVA and Bonferroni post-tests; data are expressed as mean ± standard deviation; significance = *p* ≤ 0.05. Prism version 4.00 for Windows, GraphPad Software 8 (San Diego, CA, USA) was employed. For the calculation of the inhibitory concentration 50% (IC50), the soft “Quest Graph™ IC50 Calculator” was used [24].

## 3. Results and Discussion

### 3.1. Synthesis and Characterization of Gold Nanoparticles

Goji berries, also designated as “superfruits”, are well known as a rich source of compounds with potent antioxidant capacities, which confers to these fruits important health-promoting properties, as they alleviate the negative effects of oxidative stress and prevent the damage of DNA, proteins or lipids caused by harmful free radicals [25]. The major bioactive compounds reported in goji fruits are phenolic derivatives, organic acids and carotenoids. The antioxidant capacity of the *L. barbarum* fruit extract, used in the green synthesis of gold nanoparticles, was evaluated using the ABTS radical scavenging assay, while its total phenolic content was assessed using the Folin–Ciocalteu method. The antioxidant capacity of the goji fruit extract was also evaluated using the FRAP method. The antioxidant activity of natural extracts, determined using the FRAP method, estimates the ability of antioxidants to reduce iron(III) to iron(II). The recorded high values of 714 ± 29.8 mg GAE/L and 39.3 ± 1.83 mM Trolox (ABTS) and 1.75 ± 0.07 mM Trolox (FRAP), respectively, indicated a high content of antioxidant metabolites, recommending the use of *L. barbarum* fruit extract both as a source of reducing agents for the Au^3+^ ions present in the HAuCl_4_ aqueous solution and of stabilizing mediators in the synthesis of nanoparticles containing phytocompounds capable of enhancing their biological properties on their surface, especially their antioxidant and anti-inflammatory protective effects against hyperglycemia-induced oxidative stress. The successful preparation of AuNPs was primarily confirmed visually by a color change from faint orange to dark purple-red.

**Figure 1:**
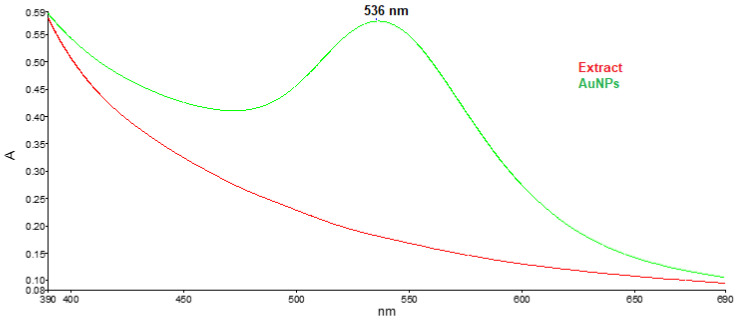
UV-vis spectra of goji fruit extract and gold nanoparticles.

In order to investigate the shape and size of the obtained nanoparticles and confirm the presence of bioactive compounds from goji berries on the surface of metal nanoparticles, the most modern spectroscopic (UV-VIS and FTIR) and microscopic (transmission electron microscopy—TEM) techniques were used. The synthesis of gold nanoparticles has been validated using UV-vis spectroscopy. Metal nanoparticles possess unique optical properties due to the resonance of the surface plasmon (SPR). In the case of gold nanoparticles, the excitation of SPR in colloidal solution is responsible for producing absorption maxima situated at wavelength values in the range of 500–550 nm [26]. Figure 1 displays the UV-vis spectra of goji fruit extract and green synthesized AuNPs, depicting the presence of the characteristic gold SPR band at 536 nm.

**Figure 2:**
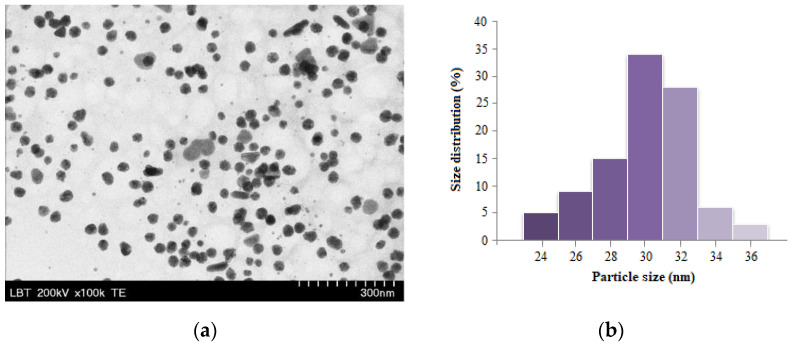
TEM image of AuNPs (**a**) and the corresponding size distribution (**b**).

The sizes and morphologies of gold nanoparticles synthesized with bioactive compounds from goji extract were analyzed using transmission electron microscopy (TEM) (Figure 2a). The TEM image confirms the presence of well-dispersed, mostly spherically shaped AuNPs, with an average diameter of 30 nm (Figure 2b).

**Figure 3:**
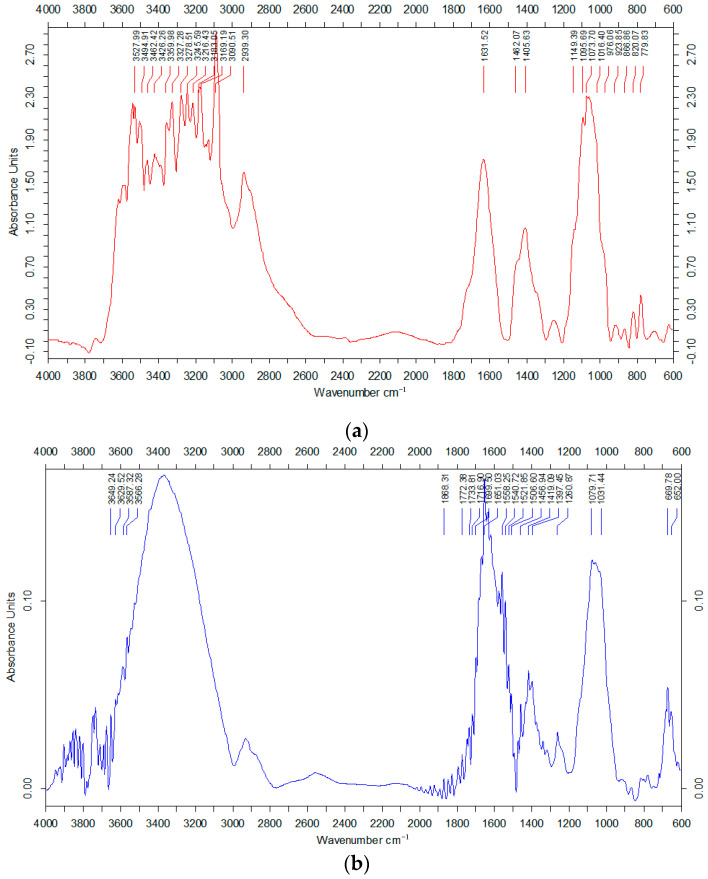
FTIR spectra of goji fruit extract (**a**) and gold nanoparticles (**b**).

FTIR analysis was applied in order to identify and confirm the presence of biomolecules from the fruit extract acting as stabilizing agents present at the surface of metal nanoparticles. The FTIR spectrum of the goji fruit extract (Figure 3a) shows a wide absorption band between 3090 and 3527 cm^−1^, characteristic of the vibration of the OH groups in the phenolic compounds. The peak situated at 1631 cm^−1^ can be attributed to the vibration of the C=O bond (carbonyl) present in the flavonoid structure. The FTIR spectrum of AuNPs (Figure 3b) showed similar absorption bands: a wide peak at 3372 cm^−1^ corresponding to the characteristic vibration of the OH groups (phenolic or alcoholic) and a band at 2936 cm^−1^, which can be attributed to the stretching vibration of C-H bond, while at 1651 cm^−1^, a peak appeared, indicating the presence of the C=O bond in the biomolecules located on the surface of the metal nanoparticles. All these absorption bands observable in the FTIR spectra support the claim that phenolic phytocompounds such as phenolic acids and flavonoids from goji berries are present on the surface of phytosynthesized metallic nanoparticles, acting as coating ligands and being responsible for the effective stabilization of synthesized hybrid bionanomaterials.

**Figure 4:**
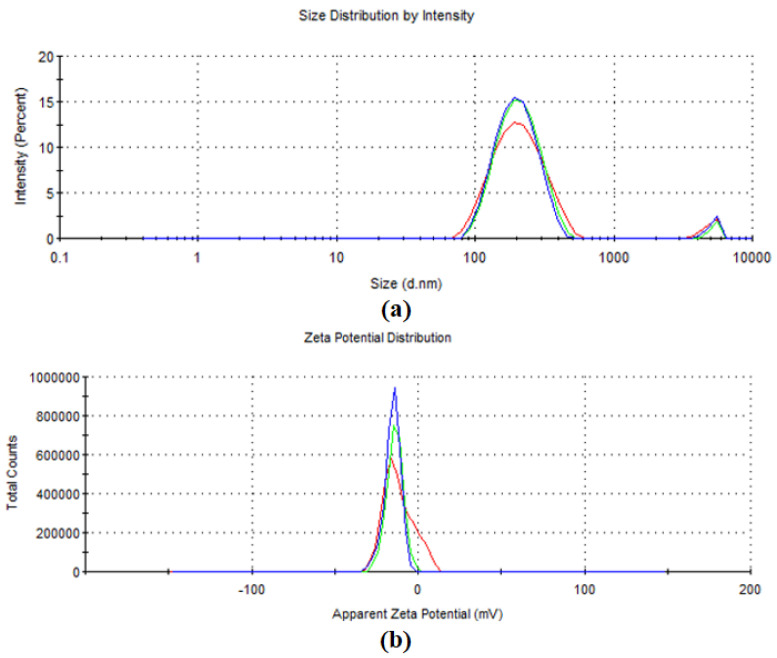
Hydrodynamic diameter (**a**) and zeta potential (**b**) of the obtained gold nanoparticles.

The stability of gold nanoparticles synthesized with biomolecules from the goji berry extract was also investigated by determining the electrokinetic potential of the obtained colloidal systems (ζ-potential). Zeta potential value (surface charge) is a measure of the electrical charge on the surface of metallic nanoparticles, being an indirect measure of their physical stability. The value of the measured zeta potential of the synthesized AuNPs (Figure 4) was −11.8 mV, suggesting good stability, while their hydrodynamic diameter was 195.8 nm, thus indicating that anionic capping agents from the goji fruit extract are coordinated to the surface of silver nanoparticles’ metallic shells.

The free radical scavenging capacity of the goji-compound-mediated synthesized gold nanoparticles was assessed using the ABTS in vitro model. The quenching ability of the ABTS free radicals was visually observed through the discoloration of the green-blueish ABTS^·+^ solution, and the antioxidant capacity of AuNPs was spectrophotometrically measured and compared to the Trolox standard. The obtained value was 3.53 ± 0.14 mM Trolox, proving the ability of bioconjugated AuNPs to neutralize the free radicals.

### 3.2. Cell Viability

**Figure 5:**
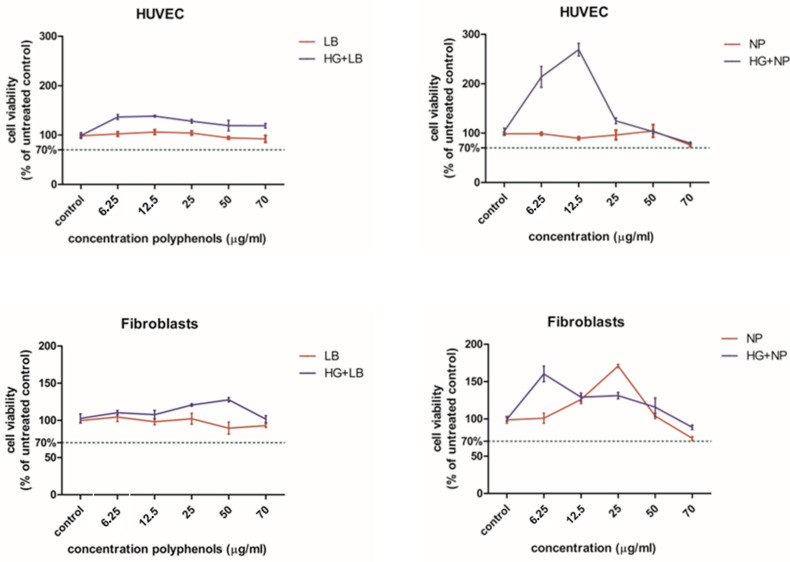
Comparative viability assessment of the cells (HUVECs—upper panels and fibroblasts—lower panels) exposed to *L. barbarum* extract and NP w/wt high glucose exposure. Data are presented as % of untreated control (media ± SD), n = 3.

The viability of the endothelial cells and fibroblasts exposed to *L. barbarum* was not affected (Figure 5). When cells were exposed to hyperglycemia and *L. barbarum* extract, the cell viability was slightly increased for all concentrations in HUVECs and for the 25 µg/mL and 50 µg/mL in fibroblasts. The proliferation inhibitory concentration 50% (IC50) was calculated for HUVECs (IC50 = 41.53 µg/mL for *L. barbarum* and IC50 = 44.22 µg/mL for HG + LB) and for BJ fibroblasts (IC50 = 35.135 µg/mL for *L. barbarum* and IC50 = 76.26 µg/mL for HG + LB). The viability of HUVECs exposed to nanoparticles was maintained above the toxicity limit at concentrations up to 50 µg/mL. The viability was decreased by 70 µg/mL to almost 70% of the untreated control. When exposed to hyperglycemia, HUVECs showed strong proliferation at low concentrations of nanoparticles (6.25 and 12.5 µg/mL). At higher concentrations, there was similar behavior to normal glucose medium. The IC50 values for HUVECs treated with AuNPs were IC50 = 78.35 µg/mL for AuNPs and IC50 = 23.76 µg/mL for HG + NP.

In the fibroblasts, nanoparticles stimulated proliferation in a dose-dependent manner, with a maximum at 25 µg/mL. At 70 µg/mL, viability was slightly above the toxicity limit. A similar effect was obtained in fibroblasts exposed to both HG and AuNPs, but the proliferation stimulation was seen at a lower AuNP concentration (6.25 µg/mL). Higher concentrations induced lower proliferation. At the maximum concentration, the viability of the fibroblasts was similar to the controls. For fibroblasts, IC50 = 52.01 µg/mL for NP, and IC50 = 57.22 µg/mL for HG + NP. Following the viability test, the concentration of 6.5 µg/mL AuNPs and, respectively, *L. barbarum* extract was chosen for the oxidative stress and inflammatory cytokine assessment.

### 3.3. Oxidative Stress

**Figure 6:**
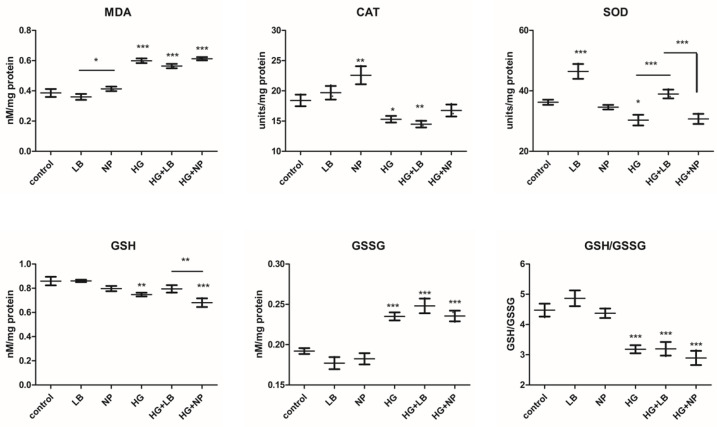
Oxidative stress assessment. MDA = malondyaldehyde, CAT = catalase, SOD = superoxide-dismutase, GSH = reduced glutathione, GSSG = oxidized glutathione. GSH/GSSG ratio was calculated. Bars represent mean ± SD (n = 3); * = *p* < 0.05, ** = *p* < 0.001, *** = *p* < 0.0001; control versus treated group, respectively, between each two selected groups. *p* < 0.05 was considered significant; only significant differences are presented.

Oxidative stress was assessed via MDA production, an indicator of the oxidative damage to the membrane lipids, the enzymatic activities of the antioxidant defense enzymes CAT and SOD and the content of GSH (reduced glutathione) and GSSG (oxidized glutathione). The GSH/GSSG ratio was calculated and represented a marker of oxidative stress (Figure 6). Malondialdehyde production was decreased by the exposure to *L. barbarum* extract and not to AuNPs. However, AuNP exposure did not increase the MDA content. In high-glucose conditions, all groups showed significantly increased MDA levels compared to the controls. *L. barbarum* did not significantly decrease the MDA production. One-way ANOVA showed the significant interaction of hyperglycemia and the increase in MDA production (*p* < 0.0001).

The antioxidant enzymes activity and catalase and superoxide dismutase activities were both significantly decreased by hyperglycemia. CAT activities were significantly increased by AuNP exposure compared to controls. HG + AuNP partially increased the CAT activity, almost to the control. SOD activity was strongly increased by *L. barbarum* extract w/wt high glucose. AuNP-treated cells showed similar SOD activity to the control in high-glucose conditions. One-way ANOVA showed a significant decreasing effect of hyperglycemia on the CAT and SOD enzymatic activities (*p* < 0.0001).

GSH content was lower in all treated groups compared to the control. The decrease was significant for HG and HG + AuNP. There was no significant difference between the HG and HG + AuNP groups. Oxidized glutathione, GSSG content, was slightly decreased by both *L. barbarum* and AuNP exposure. Hyperglycemia significantly increased the GSSG content in all groups. The ratio between reduced and oxidized glutathione levels is considered as an indicator of oxidative stress. In the *L. barbarum* group, the ratio was increased, while in the AuNP group, it was not altered. High glucose strongly diminished the antioxidant levels in all groups (*p* < 0.0001, One-way ANOVA)

Overall, *L. barbarum* extract decreased oxidative stress induced by exposure to hyperglycemia by reducing lipid peroxidation and increased the antioxidant pool with an increased GSH/GSSG ratio and antioxidant superoxide dismutase activity. Since hyperglycemia induces the production of oxygen superoxide anions, previously reported as being involved in microvasculature damage in diabetes [12,27], the increase in the SOD enzymatic activity via *L. barbarum* extract would be highly beneficial for the oxidative statuses of the cells. Moreover, GSH content was decreased via hyperglycemia as previously reported [27], but the higher level of GSH induced by the *L. barbarum* extract led to higher scavenging ROS activity, which can normalize the oxidative balance and may contribute to a sustainable metabolic change effective against ROS induced by hyperglycemia.

The AuNPs induced higher catalase activity but did not increase the antioxidant reserve and had no impact on the lipid peroxidation induced by hyperglycemia. The level of catalase is important since it neutralizes the H_2_O_2_ produced by the activity of SOD. Enhanced H_2_O_2_ levels were reported in hyperglycemia, and they promote cell cycle arrest by decreasing Cdc25C [28]. This can lead to the release of proinflammatory cytokines via NF-kB pathway activation, autophagy or necroptosis [12].

There are reports that show the importance of antioxidants such as vitamin E, which inhibits phosphokinase C activation following hyperglycemia and retinal neoangiogenesis in diabetic rats [12,27]. Also, diabetic mouse embryopathy was prevented by treatment with vitamin C, E, SOD, N-acetyl-cysteine or glutathione ethyl ester [27]. However, in patients with diabetes mellitus who underwent the administration of antioxidants, such as vitamin E 400 UI/day, for 4 years in a clinical study, Heart Outcomes Prevention Evaluation (HOPE) provided no preventive effects against cardiovascular complications, while shorter studies showed better outcomes with vitamin E supplementation on retinal neoangiogenesis or nephropathy [27]. Therefore, other types of antioxidants that increase antioxidant enzymatic activity and antioxidant reserve, such as GSH, can have a higher impact on the oxidative balance of cells, leading to a better outcome.

### 3.4. Inflammatory Cytokines

**Figure 7:**
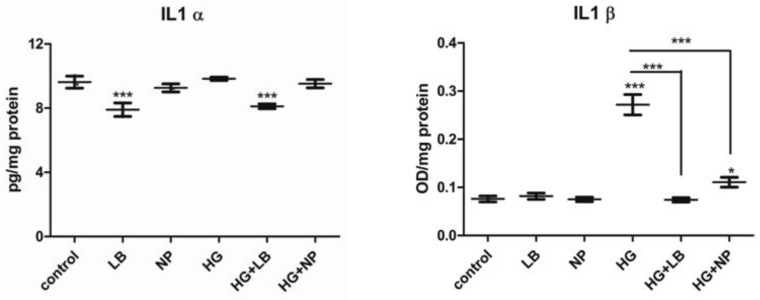
Inflammatory cytokines. *p* < 0.05 was considered significant. Bars represent mean ± SD (n = 3); * = *p* < 0.05, *** = *p* < 0.0001; control versus treated group, respectively, between each two selected groups.

IL1α production was significantly decreased by *L. barbarum* w/wt HG compared to the controls. The NP groups showed no proinflammatory response w/wt HG. High glucose induced a slight, not significant increase of IL1α level (Figure 7).

IL1β production was slightly increased by *L. barbarum*. In the high glucose group, there was a strong increase of the IL1β content, which was not seen in the HG + LB, moreover, the difference between these two groups is strongly significant. HG + NP group showed increased level of IL1β, compared to control, but strongly decreased it compared to the HG alone. These data show an anti-inflammatory effect of both *L. barbarum* extract and NP in high glucose conditions.

The cytokine family of IL1 contains 11 members with multiple roles in inflammation and malignancies. Both IL1α and IL1β use the same receptor IL1R and have proinflammatory functions. IL1α is an unusual cytokine, leading to a sterile inflammatory status in human diseases like autoimmune diseases and cancer. IL1α is expressed constitutively in epithelial and endothelial stromal cells. It can be stimulated in other cells by Toll-like receptor agonists [29], inflammatory cytokines [30,31,32] and oxidative stress [33,34,35], which is reviewed in [36]. Hyperglycemia exposure triggers the release of proinflammatory cytokines, leading to an inflammatory status, responsible for some of the complications in diabetes, such as retinopathy, neuropathy and cardiovascular diseases. Therefore, combined antioxidant and anti-inflammatory activity would be beneficial in hyperglycemic conditions to delay the onset of complications and their further aggravation. Overall, the *L. barbarum* extract, and to a lesser extent, the AuNPs synthesized using *L. barbarum* extract were able, at non-toxic, low concentrations, to decrease the proinflammatory effect of hyperglycemia, leading to the release of IL1β.

IL1α can bind AP1 and NF-kB, leading to their activation during inflammation. IL1β is mainly expressed in immune cells and also endothelial cells; its level is increased by IL1α signaling through AP1 and NF-kB. IL1β was found to be increased in patients with type 1 diabetes mellitus having poor disease therapy and also in the early stage. Moreover, increased IL1β production was responsible for the inflammatory statuses of patients with diabetes mellitus type 1 [37].

Increased IL1β levels were also reported in obese and diabetic patients (type II) and were involved in β-cell dysfunction, leading to deregulated insulin secretion and the promotion of the disease and acting as a link between immunity and metabolism. IL1β signaling induces the acute phase response, vasodilatation, fever, low blood pressure and the further activation of general inflammatory mechanisms. In hyperglycemic patients, IL1β increased levels are produced as a result of inflammasome and particularly NLRP3 signaling. The NLRP3 inflammasome is made from several proteins: a sensor (NLRP3); an adaptor (ASC)—apoptosis-associated speck-like protein—which comprises a domain for caspase recruitment; and procaspase 1, the effector, which activates IL1β and IL18 synthesis [38]. The decrease in IL1β induced by hyperglycemia as a result in cells treated with *L. barbarum* or AuNPs is an important anti-inflammatory effect leading to possible beneficial effects in both the prevention and therapy of hyperglycemia.

Both compounds tested, *L. barbarum* extract and AuNPs, were well tolerated by the HUVECs and dermal fibroblasts. Overall, in the hyperglycemia-stimulated endothelial cell model employed in the current study, *L. barbarum* extract showed important protective antioxidant effects against hyperglycemia-induced oxidative alterations. *L. barbarum* decreased lipoperoxidation, increased SOD activity and increased antioxidant balance GSH/GSSH. Moreover, proinflammatory cytokine IL1α and β levels were decreased. AuNPs synthesized using the *L. barbarum* extract partially recuperated the antioxidant activity of the SOD and especially CAT enzymes, reduced by hyperglycemia exposure and decreased IL1β, strongly augmented by hyperglycemia to almost control levels. Therefore, both compounds show promising antioxidant and anti-inflammatory effects in endothelial cells exposed to hyperglycemia.

## 4. Conclusions

The present study reports an eco-friendly, one-pot synthetic method of gold nanoparticles by effectively exploiting the antioxidant properties of *L. barbarum* fruits, which successfully acted as reducing and capping agents of the obtained nanoparticles. The obtained bioconjugated AuNPs were mostly regular in shape, spherical, with an average diameter of 30 nm.

In the endothelial cells, AuNPs showed protective effects against the oxidative stress and proinflammatory status induced by hyperglycemia, responsible for endothelial injuries, leading to several diabetes complications. AuNPs partially recuperated the antioxidant defense, particularly catalase activity, and inhibited the IL1β proinflammatory cytokine release induced by hyperglycemia. Therefore, the AuNPs retained some of the antioxidant and anti-inflammatory properties of the *L. barbarum* extract and showed promising results against hyperglycemia’s deleterious effects.

## Data Availability

The data presented in this study are contained in the article.
